# Reproductive status of male rat offspring following exposure to methamphetamine during intrauterine life: An experimental study

**DOI:** 10.18502/ijrm.v21i2.12809

**Published:** 2023-03-08

**Authors:** Zahra Khoshgoftar Some Saraii, Soroush Dianaty, Fatemeh Rouhollah, Nayereh Zare, Batool Ghorbani Yekta

**Affiliations:** ^1^Department of Cellular and Molecular Sciences, Faculty of Advanced Sciences and Technology, Tehran University of Medical Sciences, Islamic Azad University, Tehran, Iran.; ^2^Student Research Committee, Tehran Medical Sciences, Islamic Azad University, Tehran, Iran.; ^3^Universal Scientific Education and Research Network (USERN), Tehran, Iran.; ^4^Department of Anatomical Sciences and Cognitive Neuroscience, Tehran Medical Sciences Branch, Islamic Azad University, Tehran, Iran.; ^5^Department of Physiology, Faculty of Medicine, Tehran Medical Sciences, Islamic Azad University, Tehran, Iran.

**Keywords:** Methamphetamine, Testis, Fertility, Reproduction, Apoptosis, Intrauterine exposure.

## Abstract

**Background:**

Methamphetamine abuse during pregnancy is associated with maternal and fetal adverse outcomes. Methamphetamine induces reproductive damage in adults; however, its effect has not been studied during pregnancy.

**Objective:**

To investigate the effects of methamphetamine exposure during pregnancy on the reproductive system.

**Materials and Methods:**

15 pregnant Wistar rats were divided into 3 groups (n = 5/group), they received daily intraperitoneal injections of saline or methamphetamine (5, and 10 mg/kg) from day 10 until the end of pregnancy. One adult male offspring was selected from each dam. Subjects were euthanized, and their testis was removed. Sperm samples from cauda epididymis were analyzed for sperm concentration, morphology, and motility. Terminal deoxynucleotidyl transferase dUTP nick-end labeling assay was used to detect apoptotic cells. Levels of B-cell lymphoma 2 protein (Bcl-2) and Bcl-2 associated X-protein were measured using Western blot.

**Results:**

Methamphetamine significantly decreased sperm concentration (5 mg vs. saline: p = 0.001, 10 mg vs. saline: p < 0.001), normal sperm morphology (saline vs. 10 mg: p = 0.001), and motility (p: saline vs. 5 mg = 0.004, 5 mg vs. 10 mg = 0.011, saline vs. 10 mg < 0.001) in a dose-dependent manner. There was a significantly higher number of terminal deoxynucleotidyl transferase dUTP nick-end labeling -positive cells and higher exposure. Moreover, Bcl-2 associated X-protein was increased, and Bcl-2 was decreased in these rats.

**Conclusion:**

The present study shows that chronic methamphetamine exposure during intrauterine period can induce apoptosis of seminiferous tubules and decrease sperm quality in adult rats. Moreover, we showed that the intrinsic apoptotic pathway is involved in this process. Further studies are required to identify the complete molecular pathway of these results.

## 1. Introduction

Methamphetamine is a central nervous system stimulant and recreational drug thatinduces a sense of euphoria, increased energy, sexual desire, and risky sexual behavior in users (1-3). It is associated with neurological damage, cognitive dysfunction, and impaired judgment (1). Methamphetamine use during pregnancy has been associated with birth defects, low birth weight, small head circumference, and intrauterine growth restriction in humans (2) and exencephaly, premature death, and subarachnoid hemorrhage in mice (4, 5). The adverse neurological outcomes of fetal methamphetamine exposure are well documented (2, 6, 7), but the research about other organ systems is limited.

Animal experiments have documented the adverse effects of methamphetamine on the adult male testis such as increased apoptosis, decreased cell proliferation, and a gap in the epithelium between the spermatogonia and other layers (8, 9). These effects have been reported in doses as low as 4 mg/kg (10, 11).

Studies link methamphetamine to decreased sperm count, abnormal sperm morphology, and low sperm motility (10-13), in addition to decreased serum testosterone levels (8, 12).

Methamphetamine can affect apoptosis by increasing apoptotic index, oxidative stress, and cleaved caspase-3 (12). It is shown to diffuse into mitochondria and decrease membrane potential, disrupting the electrochemical gradient, and causing the release of cytochrome c from mitochondria, followed by caspase activation (1). Intrinsic apoptosis is mediated by proapoptotic proteins such as B-cell lymphoma 2 (BCL-2) associated X-protein (BAX), which increases the mitochondrial outer membrane permeability to allow the release of proteins such as cytochrome c and activating caspases (14, 15). Membrane permeabilization is considered “the point of no return” in this process, after which the cell is committed to the fate of apoptosis (14). The action of BAX is antagonized by antiapoptotic proteins such as Bcl-2, which binds to BAX. Cell shift toward apoptosis is determined by the balance between proapoptotic and antiapoptotic Bcl-2 family proteins (14). Therefore, upregulation of BAX and downregulation of Bcl-2 are considered an initial step toward apoptosis (14). Changes in apoptosis regulator proteins can result from internal or external stimuli such as DNA damage (15).

While methamphetamine has been associated with damage to the male reproductive system in adults, its effects on the fetus's reproductive system are less known. Previous articles have focused solely on methamphetamine users themselves, and their offspring have not been studied. We aimed to investigate the potential effects of chronic methamphetamine exposure on the reproductive system of children born to methamphetamine-abusing mothers. To this end, the present study evaluates the reproductivity of a generation of adult rats chronically exposed to methamphetamine in the intrauterine period.

## 2. Materials and Methods

### Animal treatment and drug administration

This was an experimental study conducted at Islamic Azad University Tehran Medical Sciences Branch, Tehran, Iran. 30 adult Wistar rats (15 male and 15 female) were procured from Pasteur Institute (Tehran, Iran). All rats were in good health and were never exposed to methamphetamine or any other intervention before. After estrus detection, female and male rats were caged together for one night, after which they were separated and the females were smeared vaginally.

The detection of vaginal plug was designated as day 1 of gestation. Female rats were randomly assigned to 3 groups (n = 5/each): the saline group received an injection of 0.9% NaCl, 1 ml/kg. The 5 mg, and 10 mg groups received an injection of methamphetamine hydrochloride (5 mg/kg and 10 mg/kg, respectively). Methamphetamine hydrochloride (synthesized and analyzed by Central Research Laboratories of Shahid Beheshti University of Medical Sciences, Tehran, Iran) diluted in normal saline was used for this experiment. The female rats were injected once per day from day 10 until the end of pregnancy. On day 20 of gestation, each pregnant rat was housed in a separate cage. All rats gave birth at days 21-22 of gestation. All offspring lived in the cage with their mother until weaning at 28 days of age, after which the male and female offspring were segregated. Each group of male offspring were housed in a separate cage. All groups were kept at the same conditions (food and water ad libitum, 12-hr light/dark cycle, and 20 C) and did not receive methamphetamine, saline, or any other intervention. One male offspring from each mother was randomly allocated to a group using computer-generated random numbers.

### Sample collection

Subjects were euthanized at 12 wk of age. The literature suggests that Wistar rats are sexually mature by the end of 10 wk (16, 17). Euthanasia was carried out by decapitation using guillotines after sedation. Rats were dissected using a midline incision, and the testes were removed. The epididymis was removed from the testis and used for sperm analysis. The testes were fixed using a 10% formalin solution and sent to a lab for analysis. Both left and right testes (or epididymis) were used for each analysis, and their average was used for each variable.

### Sperm analysis

The cauda epididymis was cut open and placed in 2 ml of phosphate-buffered saline (PBS) for a few minutes to allow its contents to disperse. A solution sample was collected and viewed under a bright-field microscope within 1 hr of obtaining the sample. The sperm concentration, morphology, and motility were examined under the microscope at 10 different random fields of view, and the average was used. Normal sperm morphology was defined using the classification by Krzanowska (18). Sperms with progressive motility and non-progressive motility were counted as normal motile sperm. The researcher carrying out the sperm analysis was blinded to the group allocations.

### Terminal deoxynucleotidyl transferase dUTP nick-end labeling (TUNEL) assay

The testes were sectioned, fixed using paraffin, and mounted on microscopic slides. TUNEL assay was carried out using Roche in Situ Cell Death Detection Kit, Fluorescein (Cat. No. 11684795910), according to the product instructions. In brief, the slides were deparaffinized in xylene and rehydrated with ethanol. Slides were then washed with PBS, incubated with Proteinase K solution at room temperature for 30 min, and washed again. The slides were incubated with a TUNEL reaction mixture for 60 min at 37 C, washed with PBS, and viewed under a fluorescence microscope. For each slide, the number of TUNEL-positive cells in 20 seminiferous tubules at 10 different microscopic fields (200 tubules for each slide) were counted. The researcher carrying out the analysis was blinded to the group allocations.

### Western blot analysis

Western blot analysis was used to measure levels of Bcl-2, and BAX. Testis tissues were dissolved in a lysis buffer consisting of 50 mM Tris-HCl (pH 8.0), 0.25% sodium deoxycholate, 0.1% sodium dodecyl sulfate (SDS), 150 mM NaCl, 1 mM Ethylenediaminetetraacetic acid, 0.1% Triton X-100, complete protease inhibitor cocktail and phosphatase inhibitors cocktail. Furthermore, 60 mcg of total protein was measured using Bradford assay and loaded into each well of the SDS- polyacrylamide gel electrophoresis apparatus. Electrophoresis was performed in a running buffer consisting of 3.0 g Tris base, 14.4 g glycine, and 1.0 g SDS in 1000 ml of H ${}_{2}$ O for 2 hr at 90 volts. The proteins on the gel were transferred to a polyvinylidene fluoride membrane, and a blotting sandwich was formed by placing filter papers and a sponge on either side of the membrane secured by a plastic frame.

The blot sandwich was placed in a blot tank filled with a transfer buffer consisting of 3.2 g Tris base, 14.2 g glycine, and 200 ml methanol in 1000 ml of H ${}_{2}$ O for 1.5 hr at 100 volts. The membranes were placed in a blocking buffer consisting of 40.0 ml Tris-buffered saline (TBS), 1.0 g skim milk, 20.0 µl tween 20- and 1.0-ml-glycerol overnight and then washed with Tris-Buffered Saline-Tween (100 ml TBS, 1 ml tween 20 in 1000 ml H ${}_{2}$ O, pH 7.5) 3 times for 5 min each. The membranes were put on a shaker with the primary antibodies for BAX (ab216494), BCL-2 (ab196495), and Glyceraldehyde 3-phosphate dehydrogenase (GAPDH) (ab181602) for 3 hr at room temperature and then put on a shaker with the secondary antibody, mouse IgG lambda binding protein conjugated to horseradish peroxidase (sc-516132), for 2 hr at room temperature. The membranes were washed with Tris-Buffered Saline-Tween 3 times for 5 min after each antibody incubation. The protein bands were detected by electrochemiluminescence on x-ray films. Quantification was performed using ImageJ 1.41o with GAPDH levels as a loading control to normalize the results. The researcher carrying out the analysis was blinded to the group allocations.

### Ethical considerations

Animal procedures used in this study were according to National Institutes of Health guidelines for the care and use of laboratory animals. This study was approved by the Research Ethics Committees of Islamic Azad Tehran Medical Sciences University - Pharmacy and Pharmaceutical Branches Faculty, Tehran, Iran (Code: IR.IAU.PS.REC.1399.230).

### Statistical analysis

The collected data was numerical which was described using mean and standard deviation. The normality of data was assessed using Kolmogorov-Smirnov test. The statistical differences between groups were tested by Student's *t* test using SPSS software (Version 25.0. Armonk, NY: IBM Corp). A p-value < 0.05 was considered statistically significant.

## 3. Results

There were 5 adult male rats in each group. The mean weights of the rats were 227.6 ± 15.17, 225.6 ± 17.27, and 224.8 ± 16.57 gr for saline, 5 mg, and 10 mg groups, respectively. No statistical difference was observed between mean group weights (p > 0.05 for all comparisons). No visible abnormalities were detected in the subjects.

**Figure 1:**
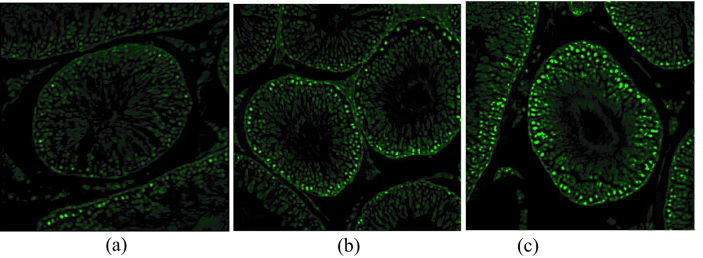
TUNEL (Terminal deoxynucleotidyl transferase dUTP nick-end labeling) assay of testis ( × 40): saline (a), 5 mg/kg (b), 10 mg/ kg (c).

**Figure 2:**
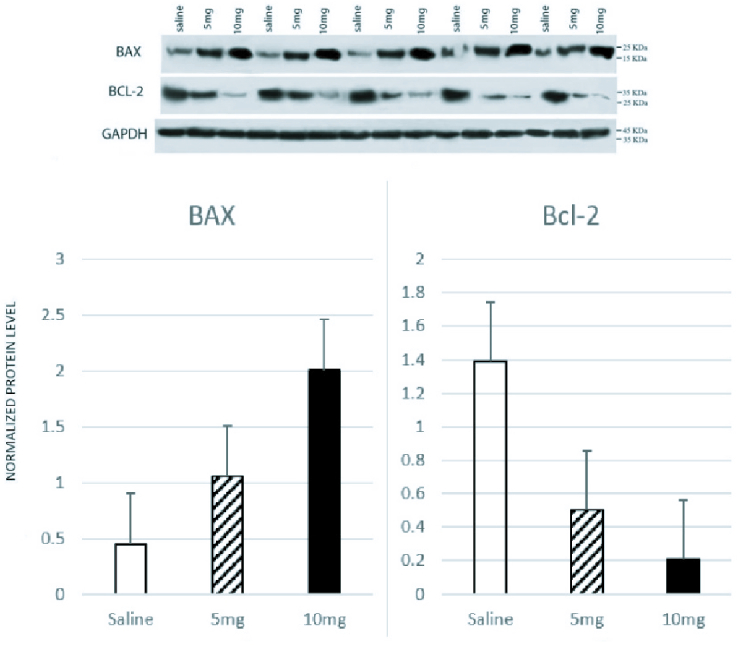
Western blot analysis (Bcl-2: B-cell lymphoma 2 protein, BAX: Bcl-associated X-protein, GAPDH: Glyceraldehyde 3-phosphate dehydrogenase).

Sperm concentrations observed in the 5 mg group and 10 mg group were significantly lower compared to the saline group (p = 0.001 and p < 0.001, respectively). The difference between the 5 mg and 10 mg groups was not statistically significant (p = 0.14). A significantly lower percentage of morphologically normal sperms was observed in the 10 mg group compared to the saline group (p = 0.001). The difference between the saline vs. 5 mg group and the 5 mg vs. 10 mg group was not statistically significant (p = 0.213 and p = 0.18, respectively). The percentage of motile spermatozoa was also lower in cases vs. the control group (p: saline vs. 5 mg = 0.004, 5 mg vs. 10 mg = 0.011, saline vs. 10 mg < 0.001). Moreover, a significant increase of apoptotic cells was seen in seminiferous tubules of rats with higher prenatal methamphetamine exposure (Figure 1; p < 0.001 for all comparisons). In Western blot analysis (Figure 2) significant differences were observed between groups in Bcl-2 and BAX levels. Compared to the control group, a significant increase was observed in the level of BAX/GAPDH in the 5 mg and 10 mg groups (p < 0.001 for all comparisons). The level of Bcl2/GAPDH was decreased in a dose-dependent manner from the saline group to the 10 mg group which was statistically significant (p: saline vs. 5 mg < 0.001, 5 mg vs. 10 mg = 0.004, saline vs. 10 mg < 0.001). The result of all comparisons is shown in table I.

**Table 1:** Sperm quality and testis apoptosis comparison between groups


**Variables**	**Saline**	**5 mg**	**10 mg**	**P-value**
**Sperm concentration ( × 10^6^/mL)**	30.62 ± 2.53	23.50 ± 1.96	16.78 ± 4.36	• Saline vs. 5 mg = 0.001 ${}^{*}$ • Saline vs. 10 mg < 0.001 ${}^{*}$ • 5 mg vs. 10 mg = 0.14
**Sperms with normal morphology (%)**	64.40 ± 13.01	53.40 ± 12.72	35.80 ± 3.70	• Saline vs. 5 mg = 0.213 • Saline vs. 10 mg = 0.001 ${}^{*}$ • 5 mg vs. 10 mg = 0.18
**Sperms with normal motility (%)**	66.40 ± 4.336	55.80 ± 3.89	47.40 ± 4.22	• Saline vs. 5 mg = 0.004 ${}^{*}$ • Saline vs. 10 mg < 0.001 ${}^{*}$ • 5 mg vs. 10 mg = 0.011 ${}^{*}$
**TUNEL-positive cells**	6.8 ± 1.79	39.2 ± 7.91	78.6 ± 7.57	< 0.001 for all comparisons ${}^{*}$
**BAX/GAPDH**	2.009	0.447	1.051	< 0.001 for all comparisons ${}^{*}$
**Bcl-2/GAPDH**	1.389	0.502	0.208	• Saline vs. 5 mg < 0.001 ${}^{*}$ • Saline vs. 10 mg < 0.001 ${}^{*}$ • 5mg vs. 10 mg = 0.004 ${}^{*}$
Data presented as Mean ± standard deviation. Student's *t* test ${}^{*}$ Significant. TUNEL: Terminal deoxynucleotidyl transferase dUTP nick-end labeling, Bcl-2: B-cell lymphoma 2 protein, BAX: Bcl-associated X-protein, GAPDH: Glyceraldehyde 3-phosphate dehydrogenase				

## 4. Discussion

The present study shows that intrauterine methamphetamine exposure in male rats can lower sperm quality and damage testis, the effects of which continue into adulthood. Methamphetamine at 5 mg/kg and 10 mg/kg decreased sperm concentration, increased the percentage of morphologically abnormal and/or immotile sperms, and induced apoptosis in the seminiferous tubules of adult male rats.

Methamphetamine is considered a threat to public health and an important drug in the field of reproductive toxicology (1, 8, 10). It is a member of a class of drugs called Amphetamines, including amphetamine, ecstasy (3,4-Methylenedioxymethamphetamine or MDMA), Methylphenidate (Ritalin), and Bupropion. Amphetamines act by inducing the release and blocking the reuptake of catecholamines, mainly in the central nervous system (2, 19). Amphetamines are thought to have a chemical structure similar to monoamine neurotransmitters (dopamine, norepinephrine, and serotonin). Therefore, they act as an agonist for monoamine transporters and block the reuptake of extracellular monoamine neurotransmitters (1, 19, 20). This, in turn, leads to an increase in these neurotransmitters in the central nervous system.

Other amphetamine-based drugs have also demonstrated effects similar to those of methamphetamine.

For example, significant DNA damage, decrease in sperm count, and motility was reported in the testis of rats exposed to intrauterine MDMA (21). Similar results were observed in adult male mice exposed to Ritalin such as a significant decrease in body weight, Leydig cells, and testosterone levels (22).

Substances used by drug-abusing pregnant mothers, such as Methamphetamine can cross the placenta and enter fetal circulation (2, 23). This transfer's most common mechanism is passive diffusion resulting from concentration gradient between maternal and fetal circulation (23). Because of the weak detoxification mechanisms in the fetus, these drugs tend to concentrate in fetal organs and gradually increase over time to the point that fetal drug levels are higher than maternal levels (24). Large concentrations of drugs have been found in the placenta and other organs such as the fetus's lung, kidney, intestines, liver, brain, and heart (25). Amphetamines and their metabolites are readily detected in the umbilical cord, placenta, and amniotic fluid of exposed fetuses (23). They can induce teratogenic and fetotoxic effects on the fetus (2, 4, 5). Amphetamines in fetal circulation are thought to have effects similar to those observed in adults, namely blocking the reuptake of monoamine neurotransmitters (26).

The fetal effects of methamphetamine exposure are not limited to direct placental transfer. Methamphetamine can inhibit the serotonin transporter and the norepinephrine transporter expressed on the placenta (with a greater affinity for norepinephrine transporter) (20). This, in turn, leads to an increase in serotonin and epinephrine in the placenta and fetal circulation. Norepinephrine and serotonin rise in mother and fetus is thought to be responsible for the adverse outcomes observed in fetal methamphetamine exposure (26). They can cause fetal and maternal hypertension, uterine and placental contraction, and decreased placental blood flow (26, 27). Methamphetamine increases maternal metabolism and decreases appetite. As a result, methamphetamine abuse during pregnancy is associated with decreased maternal body weight (28, 29). Monoamine neurotransmitters also play a pivotal role in body temperature regulation, and the dysregulation of them by methamphetamine induces hyperthermia (30). Maternal and fetal hyperthermia has been linked to damage several fetal organs, including testis (1, 8).

The apoptosis-inducing effects of methamphetamine are well known. However, the exact underlying mechanism is a complicated and multifactorial process. Oxidative damage caused by the increased dopamine, norepinephrine, and serotonin is thought to play an important part in cellular apoptosis. Dopamine is rapidly converted to reactive oxygen species (ROS), including superoxide radicals, hydroxyl radicals, and hydrogen peroxide (1). In addition to increasing ROS production, methamphetamine can disrupt antioxidant systems that counteract the oxidative damage of ROS. Animal experiments have shown a decrease in CuZnSOD, catalase, and glutathione levels in the brain after methamphetamine administration. Oxidative stress has been shown to result in DNA damage and increased release of calcium ions from the endoplasmic reticulum that can induce apoptosis (1).

We assessed the involvement of the mitochondrial pathway of apoptosis in methamphetamine-induced damage by measuring the expression of BAX and Bcl-2 in the testis. Previous studies have also confirmed the role of amphetamines in increasing proapoptotic proteins and decreasing antiapoptotic proteins, which is consistent with our results (1, 12).

This study was limited to the visible testis tissue and sperm damage. Aside from BAX and Bcl-2 as indicators of the intrinsic apoptotic pathway, alternative mechanisms were not investigated. Namely, we did not measure dopamine, serotonin, and norepinephrine levels in maternal or fetal circulation. The levels of ROS were not assessed either. There was no measurement of maternal metabolism or weight loss. Hormones such as testosterone were not measured. Body temperature was not assessed to test for hyperthermia.

There was also no control group without injection in this study. However, this is one of the first studies to discuss the long-term effects of intrauterine methamphetamine exposure. Most of the research about the offspring of methamphetamine-abusing mothers revolve around defects presenting at birth and/or neurological symptoms. This study sought to address this issue by assessing the reproductive system of adult mice whose mothers were chronically exposed to methamphetamine in a dosage consistent with the existing literature. However, these results should be treated with caution when generalizing to other species, including humans.

## 5. Conclusion

In conclusion, we showed that chronic exposure to methamphetamine in the intrauterine period can induce reproductive damage in adult male rats. We also showed that the intrinsic mitochondrial pathway is one of the mechanisms responsible for this by causing apoptosis. The widespread abuse of amphetamines by pregnant women remains a global health issue. There are also concerns about the effects of prescription amphetamine-based drugs such as Ritalin. Moreover, most of the available antidepressant drugs act similar to amphetamines by blocking the functions of the serotonin transporter and the norepinephrine transporter. This warrants further research on the reproductive toxicity of these drugs, their underlying mechanisms, and longtime observation of children with intrauterine exposure to them.

##  Conflict of Interest

The authors declare that there is no conflict of interest. 
